# Decurarization After Thoracic Anesthesia using sugammadex compared to neostigmine (DATA trial): a multicenter randomized double-blinded controlled trial

**DOI:** 10.1186/s44158-024-00146-6

**Published:** 2024-02-08

**Authors:** Federico Piccioni, Giulio L. Rosboch, Cecilia Coccia, Ilaria Donati, Paolo Proto, Edoardo Ceraolo, Federico Pierconti, Martina Pagano, Daniele Vernocchi, Franco Valenza, Giorgio Della Rocca

**Affiliations:** 1 Department of Anesthesia and Intensive Care, IRCCS Humanitas Research Hospital, Rozzano, Milan Italy; 2 Anesthesia and Intensive Care Unit, IRCCS Fondazione Istituto Nazionale Dei Tumori, Milan, Italy; 3 Anesthesia and Intensive Care, Dipartimento Di Anestesia, Rianimazione Ed Emergenze AOU Città Della Salute E Della Scienza, Turin, Italy; 4 Anesthesiology and Intensive Care Unit, IRCCS Regina Elena National Cancer Institute, Rome, Italy; 5 Anesthesia Unit, AUSL Modena Area Sud, Modena, Italy; 6 School of Anesthesia and Intensive Care, University of Milan, Milan, Italy; 7 School of Anesthesia and Intensive Care, Humanitas University, Milan, Italy; 8 Department of Oncology and Oncohematology, University of Milan, Milan, Italy; 9 Department of Medical Area, University of Udine, Udine, Italy

**Keywords:** Anesthesia, Neostigmine, Neuromuscular blockade, Postoperative complications, Sugammadex, Thoracic surgery

## Abstract

**Background:**

Thoracic surgery is a high-risk surgery especially for the risk of postoperative pulmonary complications. Postoperative residual paralysis has been shown to be a risk factor for pulmonary complications. Nevertheless, there are few data in the literature concerning the use of neuromuscular blocking agent antagonists in patients undergoing lung surgery.

**Methods:**

Seventy patients were randomized in three Italian centers to receive sugammadex or neostigmine at the end of thoracic surgery according to the depth of the residual neuromuscular block. The primary outcome was the time from reversal administration to a train-of-four ratio (TOFR) of 0.9. Secondary outcomes were the time to TOFR of 1.0, to extubation, to postanesthesia unit (PACU) discharge, postoperative complications until 30 days after surgery, and length of hospital stay.

**Results:**

Median time to recovery to a TOFR of 0.9 was significantly shorter in the sugammadex group compared to the neostigmine one (88 vs. 278 s — *P* < 0.001). The percentage of patients who recovered to a TOFR of 0.9 within 5 min from reversal administration was 94.4% and 58.8% in the sugammadex and neostigmine groups, respectively (*P* < 0.001). The time to extubation, but not the PACU stay time, was significantly shorter in the sugammadex group. No differences were found between the study groups as regards postoperative complications and length of hospital stay. The superiority of sugammadex in shortening the recovery time was confirmed for both deep/moderate and shallow/minimal neuromuscular block.

**Conclusions:**

Among patients undergoing thoracic surgery, sugammadex ensures a faster recovery from the neuromuscular block and earlier extubation compared to neostigmine.

**Supplementary Information:**

The online version contains supplementary material available at 10.1186/s44158-024-00146-6.

## Background

Major surgery is known to increase the risk associated with postoperative morbidity and mortality [1]. Risk factors for postoperative complications include the complexity and duration of the procedure, the patient's underlying condition as well as failure to antagonize neuromuscular blocking drugs at the end of surgery [1, 2].

Thoracic surgery is considered a complex procedure with a high risk of postoperative pulmonary complications, which have an incidence of 14.5–40% [3–6].

Although thoracic surgery is an important risk factor for the occurrence of postoperative pulmonary complications, there is limited literature on the use of neuromuscular blocking agent (NMBA) reversals and the incidence of postoperative complications related to postoperative residual curarization (PORC). A study on PORC in patients transferred to the recovery room after surgery found that those who underwent thoracic surgery were more likely to experience critical respiratory events [7]. These data underscore the need for optimal anesthesiologic management of such patients, which also involves the proper and improved management of neuromuscular blockade at the end of surgery.

Neuromuscular blockade is crucial in thoracic anesthesia to aid endobronchial intubation and prevent coughing and diaphragmatic movements during surgery. The diaphragm muscle exhibits greater resistance to neuromuscular blockers and recovers earlier than the thumb adductor muscle, which is routinely monitored during general anesthesia [8]. A more rapid recovery of the first twitch of four during train-of-four (TOF) stimulation has also been reported during one-lung ventilation (OLV) compared with patients on two-lung ventilation with the consequence that it may be necessary to increase the total dosage of muscle relaxant during OLV [9].

To reduce the prolonged recovery period from neuromuscular blockade, a rapid and complete reversal at the end of surgery may be essential. Several studies in different settings have shown that recovery from rocuronium-induced neuromuscular blockade is significantly faster after administration of sugammadex than neostigmine, both when administered at moderate and deep blockade levels [10–12].

The aim of this study was to compare the rapidity of action of sugammadex and neostigmine in antagonizing rocuronium-induced neuromuscular blockade at the end of thoracic surgery. The study also aims to explore whether sugammadex, compared to neostigmine, may be associated with fewer side effects during recovery and fewer postoperative complications up to 30 days following surgery.

## Methods

This multicenter, randomized, double-blind, parallel group study enrolled patients scheduled for the following elective pulmonary resection: wedge, lobectomy, bilobectomy, pneumonectomy, bullectomy, or pleurodesis. Inclusion criteria aimed to select ASA I–II, cooperative adult patients (18–70 years old) with a body mass index (BMI) between 18 and 30 kg/m^2^. Exclusion criteria were as follows: pregnancy, scheduling for esophagectomy, thoracectomy, vascular resection, chronic obstructive pulmonary disease (COPD) gold classes IIIe–IV, respiratory infection, asthma, preoperative forced expiratory volume in 1 s (FEV1) < 60% of predicted, forced expiratory volume in 1 s/forced vital capacity ratio (FEV1/FVC) < 70%, preoperative diffusion lung capacity for carbon monoxide/alveolar volume ratio (DLCO/VA) < 60% of predicted, preoperative oxygen saturation (SpO_2_) < 92% or partial pressure of oxygen in arterial blood/fraction of inspired oxygen (PaO_2_/FiO_2_) ratio < 300, cardiovascular disease with metabolic equivalent of tasks (METS) score less than 4, neuromuscular disorder and kidney failure defined as estimated glomerular filtration rate (eGFR) < 30 ml/min/1.73 m^2^, core temperature < 35 °C, or palm temperature < 32 °C at end of operation.

The study was approved by the Independent Ethics Committee of the Fondazione IRCCS Istituto Nazionale dei Tumori of Milan (Italy) and by the Italian Medicines Agency (AIFA). It was also registered, prior to patient enrollment, at ClinicalTrials.gov (NCT02256280). All patients were recruited by the study staff and signed the written informed consent in three Italian thoracic centers.

### Protocol and measurements

Anesthesia management followed a standardized protocol reported in the Additional file. Neuromuscular monitoring was performed using TOF-Watch SX accelerometer system (Organon Teknika BV, Boxtel, Holland) with data recording on a personal computer using TOF-Watch SX Monitor software. The calibration procedure of the TOF-Watch SX Monitor was performed according to a standard protocol reported in the Additional file.

The anesthesiologist was free to adjust the dose of rocuronium for induction of muscle paralysis at induction of anesthesia and during surgery. At the end of the surgery, patients were randomly assigned to two groups, and the reversal of neuromuscular blockade was managed before extubation as follows:

## Sugammadex group

A. If post-tetanic count (PTC) = 1–15 sugammadex 4 mg/kg was administered.
B. If at least one twitch at the train-of-four stimulation sugammadex 2 mg/kg was given.

## Neostigmine group

A. If PTC = 1–15: Neostigmine 0.07 mg/kg together with atropine 0.02 mg/kg were administered.
B. If at least one twitch at the train-of-four (TOF) stimulation: Neostigmine 0.05 mg/kg together with atropine 0.02 mg/kg were given.

The choice not to establish a fixed residual block level to be reached before administering reversal (i.e., deep, moderate or shallow block) was made with the intention of proposing a pragmatic study protocol and as close as possible to actual clinical practice. The patient’s recovery from anesthesia until the tracheal extubation was initiated upon reaching a TOF ratio (TOFR) of 0.9 and continuing neuromuscular monitoring until the appearance of the patient’s spontaneous movements compromised accurate measurements.

Achieving a TOFR of 0.9 and 1.0 was defined by recording three consecutive values ≥ to 0.9 and/or 1.0, respectively. The possible detection of TOFR values less than 0.8 (3 consecutive measurements) after reaching a value of at least 0.9 was considered an indication of re-curarization.

### Outcomes

The primary outcome was the time from reversal administration to at least 3 TOFR values = or > 0.9. Secondary outcomes were as follows: time from reversal administration to at least 3 TOFR values = or > 1.0, time from reversal administration to tracheal extubation, muscular weakness incidence after extubation (measured by the tongue depressor test and swallow ability), hypoxemia or hypercapnia incidence, adverse events and postoperative complications incidence, length of hospital stay, and incidence of medical and surgical complications at 30 days after surgery. Definitions adopted for complications are listed in the Additional file.

Each patient was evaluated by an investigator blinded to the randomization arm during PACU stay, 2 h after discharge to the ward and on postoperative days 1, 2, 3, 4, and 5 and the day of discharge. A final visit was performed by anesthesiologists the day of discharge in order to summarize the clinical course of the patients. Follow-up at 30 days after the intervention was performed by telephone by the investigators.

Complications were coded in a standardized way using MedDRA terminology (Medical Dictionary for Regulatory Activities — http://www.meddra.org) indicating codes related to HLT (high-level Term) and PT (preferred term) hierarchical levels. At discharge and at the 30-day follow-up call, patients were also classified according to the Clavien-Dindo classification of surgical complications [13].

### Sample size

We planned to enroll 127 patients per group to detect a difference between the two reversal drugs of 5 min in achieving a TOFR = 0.9 [14], to achieve 90% power with a type I error of 0.05, employing a two-tailed t test for two samples with different standard deviation. Considering a drop-out rate of 5%, we planned to enroll 12 more patients for a total of 266 patients (133 per group).

Of 11 centers involved in the study, 8 did not actively participate in recruitment due to organizational or formal problems with regard to taking out supplementary insurance (even if guaranteed by the study sponsor). Thus, only three centers conducted the study, which was prematurely terminated in 2020 by the scientific committee because it was deemed very difficult to reach the established sample size due to the overall slow recruitment rate and the onset of the COVID-19 pandemic, as was the case for many studies worldwide [15]. At the data analysis stage, although unusual, two post hoc analyses were performed to calculate the conditional power and power of the study in relation to the number of patients recruited. These analyses showed that a statistically significant difference in the primary endpoint would have been highly unlikely if enrollment had continued to 266 cases. The details of these analyses are given in the Additional file.

Patients were randomly assigned to receive sugammadex or neostigmine according to a computer-generated randomization list with a fixed block size of 20 and a ratio of 1:1 generated by the study statistician using SAS software (version 9.22 — SAS Institute Inc.; Cary, NC, USA). Each center received a blocked randomization list every 20 recruited subjects. Allocation concealment was based on sequentially numerated opaque sealed envelopes. At the end of surgery, an anesthesiologist, not involved in the patient’s management, opened the sequentially numbered envelope containing the randomization assignment and prepared the reversal drug dose according to the protocol in a 20-ml syringe. During the surgical procedure, the anesthesiologist was given a syringe without knowledge of its contents and had to administer it within a 5-s time frame, followed by a rapid bolus of 10 ml of saline. The treatment was blinded to patients, anesthesiologists, and surgeons throughout the procedure and postoperative outcome assessment.

### Statistical analysis

Continuous variable distribution was verified with the Shapiro–Wilk test and reported as median [interquartile range — IQR]. Comparison between groups was performed using the Mann–Whitney *U*-test or Student’s *t*-test. Discrete variables are reported as numbers and percentages. Fisher’s exact test was used for the comparison of categorical variables. A sensitivity analysis was performed based on the depth of the neuromuscular blockade before the administration of the reversal agent: deep/moderate block (PTC > 0 or the measurement of maximum 3 twitches at the TOF stimulation) and shallow/minimal block (at least 4 twitches at the TOF stimulation or a TOFR < 0.9). A further analysis was performed dividing the patients in subgroups based on the anesthesia (inhaled vs. total intravenous) and surgical technique (open thoracotomy vs. video-assisted technique).

A *P*-value less than 0.05 was considered as statistically significant. All analyses were performed with SPSS 19 (IBM, Armonk, NY, USA). Figures were drawn with GraphPad Prism 6.0 (GraphPad Software, Boston, MA, USA). The trial was conducted in accordance with the original protocol. The manuscript was edited according to the CONSORT statement recommendations [16].

## Results

**Fig. 1:**
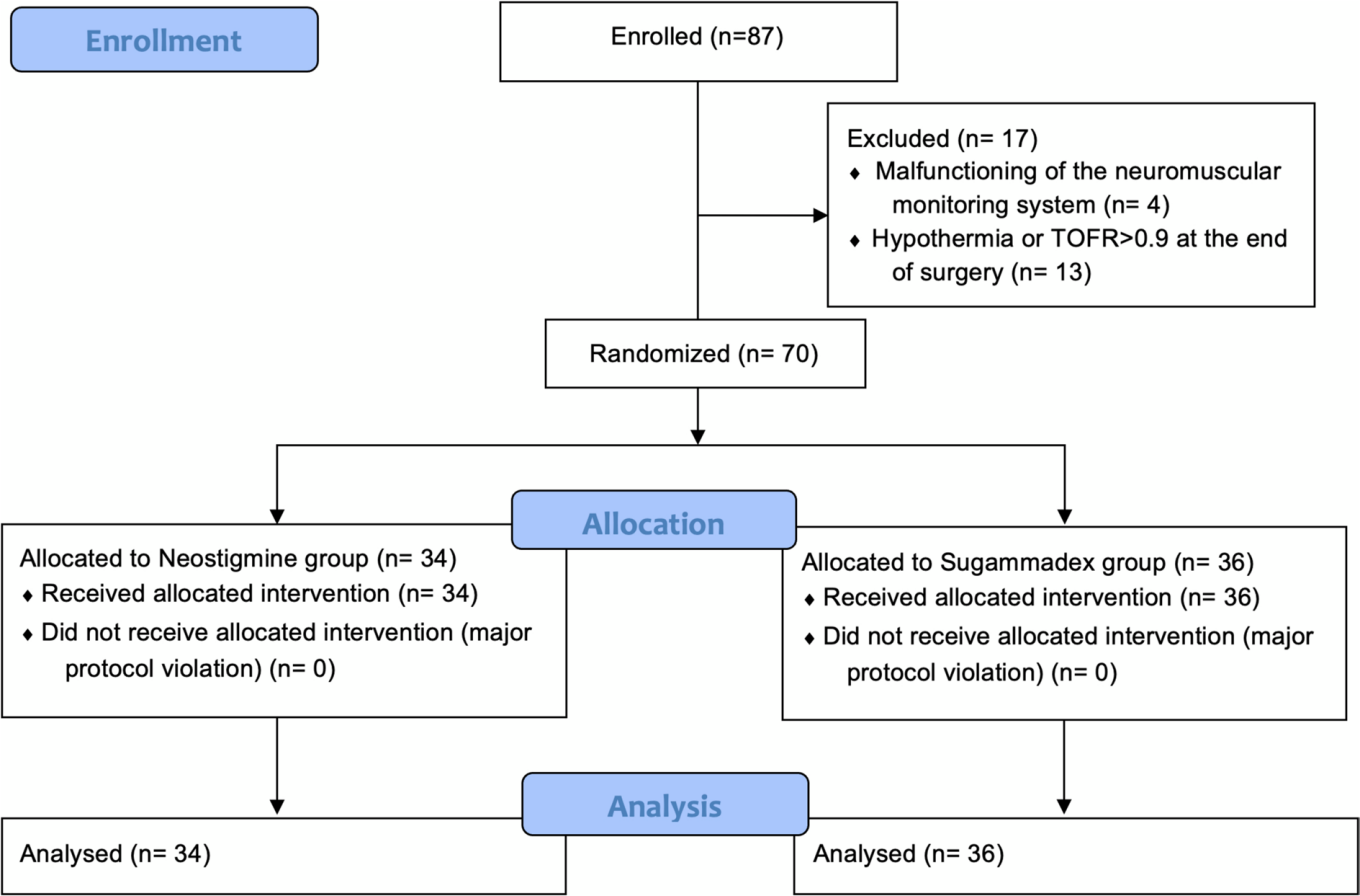
CONSORT flow chart

Eighty-seven patients were enrolled in the study between January 2015 and November 2019. Seventeen out of 87 patients were not randomized because of malfunctioning of the TOF-Watch SX Monitor (4 cases) and for clinical reasons (TOFR equal or more than 0.9 or hypothermia — Fig. 1). Overall, 70 patients, 34 in the neostigmine group and 36 in the sugammadex group, were randomized and included in the analysis. Baseline characteristics and intraoperative data of both groups are reported in Tables 1 and 2.

**Table 1:** Baseline patients’ characteristics

	Neostigmine group (*n* = 34)	Sugammadex group (*n* = 36)
Age (years)	53 (48–62)	60 (56–64)
Gender (male)	17 (50.0%)	20 (55.6%)
Height (cm)	167 (164–179)	170 (163–177)
Weight (kg)	70 (61–81)	71 (64–79)
BMI (kg.m^−2^)	25.0 (22.5–27.6)	25.0 (22.4–26.3)
Active smokers	8 (23.5%)	7 (19.4%)
Past smokers	12 (35.3%)	18 (50.0%)
ASA classes I–II	32 (94.1%)	35 (97.2%)
FEV_1_ (% of predicted value)	97 (88–107)	99 (89–109)
FEV_1_/FVC (%)	80 (79–86)	79 (73–84)
DLCO (% of predicted value)	79 (74–82)	74 (64–83)
DLCO/VA (% of predicted value)	90 (82–98)	89 (77–95)
PaO_2_ (mmHg)	80 (82–98)	89 (77–95)

Data expressed as median (IQR) or number (%). *BMI *Body mass index, *ASA *American Society of Anesthesiologists, *FEV*_*1 *_Forced expiratory volume in the first second, *FVC *Forced vital capacity, *DLCO *Diffusion capacity of carbon monoxide, *VA *Alveolar volume, *PaO*_*2 *_Partial arterious pressure of oxygen

**Table 2:** Intraoperative data

	Neostigmine group (*n* = 34)	Sugammadex group (*n* = 36)
Type of anesthesia		
Total intravenous anesthesia	27 (79.4%)	30 (83.3%)
Balanced inhaled anesthesia	7 (20.6%)	6 (16.7%)
Rocuronium dose at induction (mg)	50 (40–50)	50 (40–50)
Number of additional doses of rocuronium administered	3 (2–4)	4 (2–5)
Additional dosage of rocuronium administered (mg)	22 (20–40)	35 (20–60)
Total rocuronium dose (mg)	70 (60–90)	76 (60–115)
Neostigmine dose (mg)	3.5 (3.0–4.0)	-
Neostigmine dose (mg/kg)	0.05 (0.048–0.05)	-
Atropine dose (mg/kg)	0.02 (0.019–0.021)	-
Sugammadex dose (mg)	-	150 (140–180)
Sugammadex dose (mg/kg)	-	2.02 (2.00–2.11)
Length of anesthesia (min)	196 (147–227)	192 (142–240)
Length of surgery (min)	122 (76–154)	108 (80–146)
Surgical technique		
Thoracotomy	13 (38.2%)	13 (36.1%)
Video-assisted thoracoscopy	21 (61.7%)	23 (63.9%)
Side of surgery (right)	19 (55.9%)	23 (63.9%)
Type of surgery		
Major resection	12 (33.3%)	19 (52.8%)
Minor resection	22 (66.7%)	17 (47.2%)

Data expressed as median (IQR) or number (%)

**Fig. 2:**
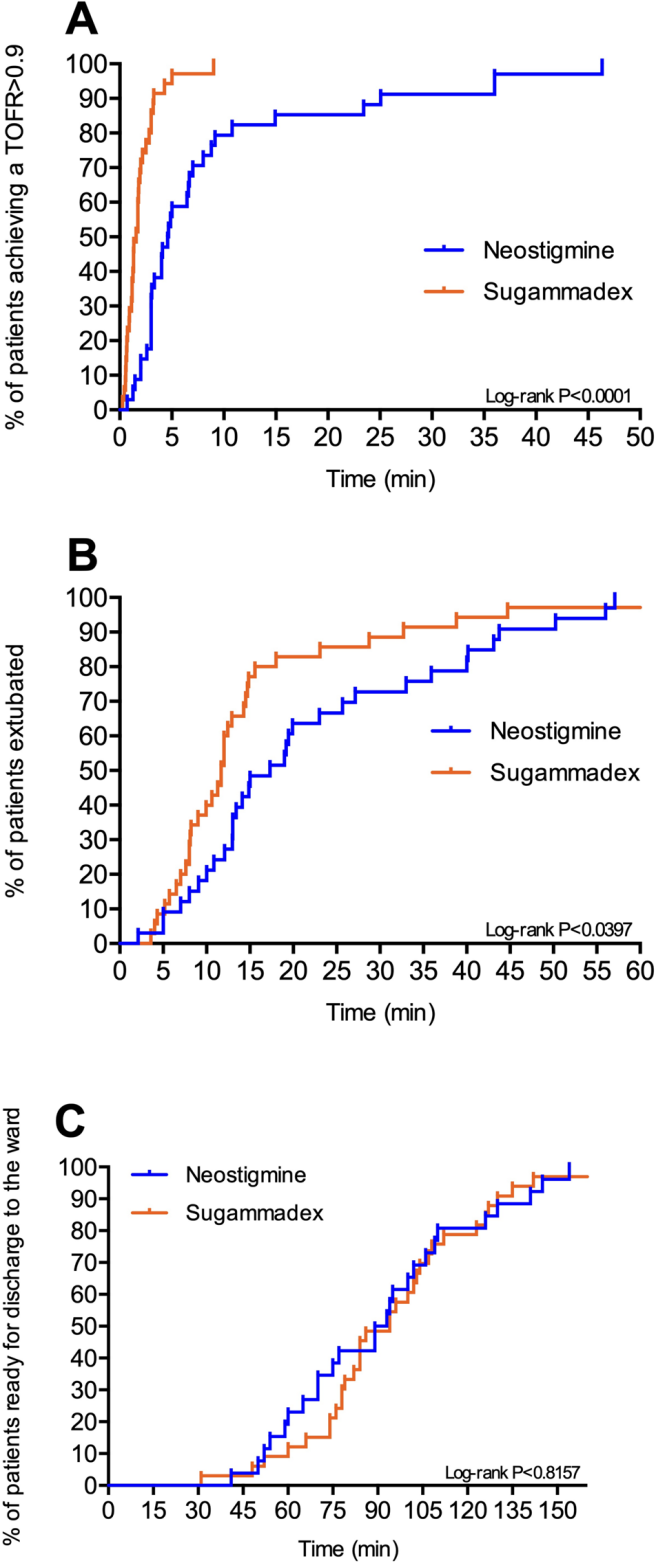
Percentage of patients achieving a train-of-four ratio of 0.9 (**A**), extubated (**B**), and discharged from the PACU (**C**) over time in the two study groups (sugammadex — red line, neostigmine — blue line)

The time from administration of reversal agents to recovery of TOFR to 0.7, 0.8, 0.9, and 1.0 was significantly faster in the sugammadex group (Table 3 and Fig. 2). Similarly, the time from reversal administration to extubation was faster in the sugammadex group (Table 3 and Fig. 2).

**Table 3:** Time from administration of reversal agents to recovery of TOFR to 0.7, 0.8, 0.9, and 1.0

	Neostigmine group (*n* = 34)	Sugammadex group (*n* = 36)	*p*-value
**Primary endpoint**			
Recovery of TOFR to 0.9			
Number of patients	34 (100%)	36 (100%)	
Median [IQR], s	278 [180–532]	88 [46–145]	< 0.001
**Secondary endpoints**			
Recovery of TOFR to 0.7			
Number of patients	30 (88.2%)	29 (80.6%)	
Median [IQR], s	149 [102–304]	55 [33–76]	< 0.001
Recovery of TOFR to 0.8			
Number of patients	30 (88.2%)	27 (75.0%)	
Median [IQR], s	195 [119–380]	80 [44–109]	< 0.001
Recovery of TOFR to 1.0			
Number of patients	18 (52.9%)	31 (86.1%)	
Median [IQR], s	358 [220–514]	121 [81–192]	< 0.001
Recovery of TOFR to 0.9 within 5 min from reversal administration	20 (58.8%)	34 (94.4%)	< 0.001
Recovery of TOFR to 0.9 within 10 min from reversal administration	28 (76.5%)	36 (100%)	0.002
Recovery of TOFR to 1.0 within 10 min from reversal administration	20 (64.5%)	36 (100%)	< 0.001
Time to extubation			
Median [IQR], min	17.3 [11–34]	11.7 [8–15]	0.011
Time to discharge to the ward			
Median [IQR], min	91 [64109]	94 [77–110]	0.572

Data expressed as median [IQR] or number (%)

No difference was found between the two groups as regards the tongue depressor test and the swallowing test (Table 4). No patient experienced severe desaturation episode after extubation. During PACU stay, pulse oximetry and respiratory rate were similar in the two study groups (Table 4). Heart rate and mean arterial pressure were higher in the sugammadex group compared to the neostigmine group. Blood gas analysis values were similar between the two groups before the discharge from the PACU. Median arterial oxygen partial pressure was 86 [76–137] mmHg and 95 [77–131] mmHg in neostigmine and sugammadex group, respectively (*P* = 0.773). Similarly, median arterial carbon dioxide partial pressure was 43 [40–47] mmHg and 43 [39–49] mmHg in neostigmine and sugammadex group, respectively (*P* = 0.600). No serious adverse events associated with sugammadex or neostigmine/atropine administration were observed in either study group.

**Table 4:** Postoperative outcomes

	Neostigmine group (*n* = 34)	Sugammadex group (*n* = 36)	*p*-value
Positive tongue depressor test^a^	2 (5.9%)	5 (13.9%)	0.429
Positive swallowing test°	2 (5.9%)	4 (11.1%)	0.674
Desaturation after extubation (SpO2 < 90%)	0 (0%)	5 (13.9%)	0.054
PONV	2 (5.9%)	6 (16.7%)	0.261
Discharge to the ward with oxygen therapy	18 (52.9%)	22 (61.1%)	0.636
All respiratory complications	5 (14.7%)	5 (13.9%)	1.000
Acute respiratory insufficiency	1 (2.9%)	0 (0%)	0.486
Pneumothorax after chest tube removal	0 (0%)	1 (2.8%)	1.000
Pleural effusion	2 (5.9%)	3 (8.3%)	1.000
Atelectasis	1 (2.9%)	0 (0%)	0.486
Pneumonia	0 (0%)	1 (2.8%)	1.000
Chylothorax	1 (2.9%)	0 (0%)	0.486
Atrial fibrillation	0 (0%)	2 (5.5%)	0.493
Chest tube days	4 [2–5]	3.5 [3, 4]	0.990
Length of hospital stay (days)	5 [3.5–6]	4 [4, 5]	0.431
Clavien-Dindo grade at discharge			
Grade 1	32 (94.1%)	32 (88.9%)	
Grade 2	2 (5.9%)	3 (8.3%)	
Grade 3	0 (0%)	1 (2.8%)	
Clavien-Dindo grade at 30 days after surgery			
Grade 1	31 (91.2%)	30 (83.3%)	
Grade 2	3 (8.8%)	1 (2.8%)	
Grade 3	0	1 (2.8%)	
Data not reported	0	4 (11.1%)	

^a^Defined by the ability (negative test) or not (positive test) to hold the tongue depressor between the teeth at the experimenter’s pull

°Defined by the subjective ability (negative test) or not (positive test) to swallow 5 ml of water

Postoperative outcomes data were not different between neostigmine and sugammadex group and are reported in Table 4. In both groups, no patient was admitted to the ICU at the end of surgery, either after extubation or in the postoperative period. No deaths occurred during the intrahospital period and at 30 days after surgery.

### Sensitivity analysis

**Fig. 3:**
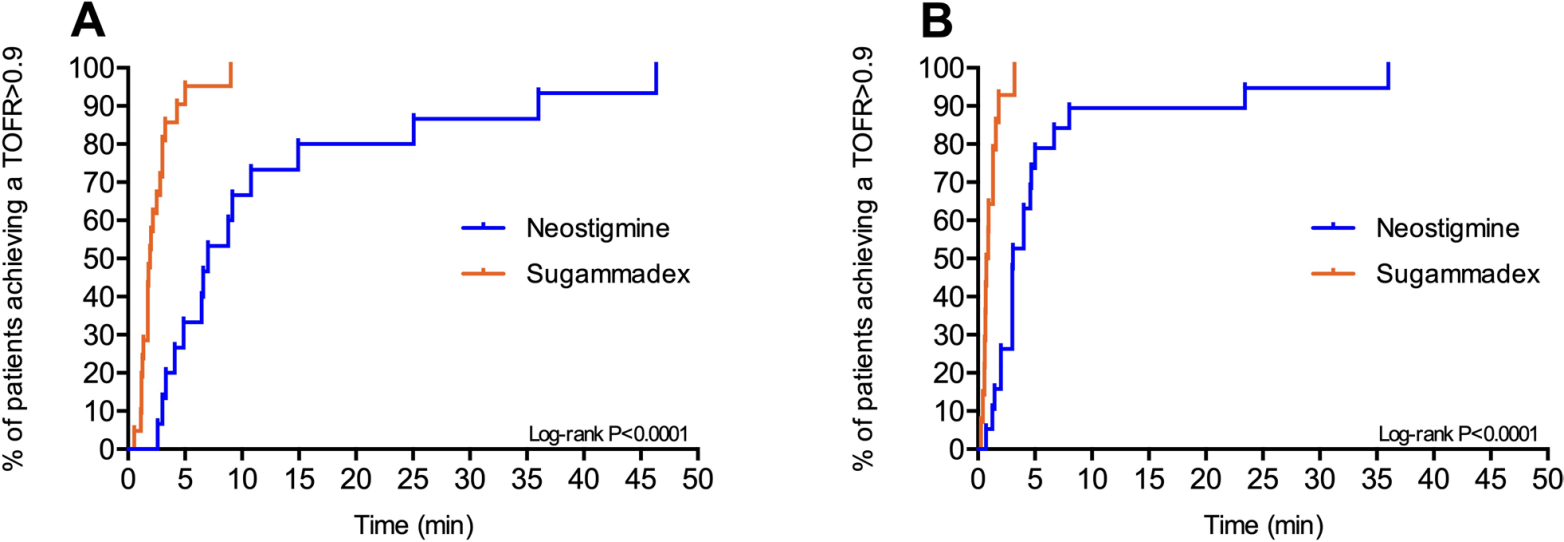
Percentage of patients achieving a train-of-four ratio of 0.9 over time in the two study groups (sugammadex — red line, neostigmine — blue line) according to the moderate/deep (**A**) or shallow/minimal (**B**) residual block subgroups

Among patients with a deep or moderate residual neuromuscular blockade at the end of surgery (36 cases), the TOFR recovery to 0.9 and the time to extubation were significantly faster in the sugammadex group than the neostigmine group (Table 5 and Fig. 3). Similar data were found among patients with a shallow/minimal block (34 subjects). No difference between the two study groups was found as regard the time from reversal administration to the discharge from the PACU in both moderate/deep and shallow/minimal residual block subgroups.

**Table 5:** Time from administration of reversal agents to recovery of TOFR to 0.7, 0.8, 0.9, and 1.0 in patients with a deep/moderate or shallow/minimal block

	Neostigmine group (*n* = 34)	Sugammadex group (*n* = 36)	*p*-value
**Deep/moderate block subgroup**			
Recovery of TOFR to 0.9			
Number of patients	15 (100%)	21 (100%)	
Median (IQR), s	420 (246–896)	115 (78–180)	< 0.001
Recovery of TOFR to 1.0			
Number of patients	9 (88.2%)	16 (80.6%)	
Median (IQR), s	472 (306–688)	151 (101–208)	< 0.001
Time to extubation			
Median (IQR), min	23 (17–43)	12 (7–15)	0.004
Time to discharge to the ward			
Median (IQR), min	93 (85–109)	102 (76–123)	0.362
Recovery of TOFR to 0.9 within 5 min from reversal administration	5 (33.3%)	19 (90.5%)	0.001
Recovery of TOFR to 0.9 within 10 min from reversal administration	10 (66.7%)	21 (100%)	0.008
Recovery of TOFR to 1.0 within 10 min from reversal administration	7 (53.8%)	21 (100%)	0.001
**Shallow/minimal block subgroup**			
Recovery of TOFR to 0.9			
Number of patients	19 (88.2%)	15 (75.0%)	
Median (IQR), s	184 (121–300)	44 (35–80)	< 0.001
Recovery of TOFR to 1.0			
Number of patients	9 (100%)	15 (100%)	
Median (IQR), sec	229 (119–452)	86 (41–140)	0.003
Recovery of TOFR to 0.9 within 5 min from reversal administration	15 (78.9%)	15 (100%)	0.084
Recovery of TOFR to 0.9 within 10 min from reversal administration	16 (84.2%)	15 (100%)	0.162
Recovery of TOFR to 1.0 within 10 min from reversal administration	13 (72.2%)	15 (100%)	0.036
Time to extubation			
Median (IQR), min	13.2 (8.6–20.6)	11.1 (8.2–15)	0.011
Time to discharge to the ward			
Median (IQR), min	89 (62–119)	84 (75–101)	0.572

*TOFR *Train-of-four ratio

Among patients managed with inhaled anesthetics, the recovery of TOFR to 0.9 was faster in the sugammadex group (135 [95–135] s vs. 292 [184–2160] s — *P* = 0.014). Similarly, a difference between the two groups was found in patients managed with total intravenous anesthesia (80 [40–119] s vs. 246 [180–527] s in sugammadex and neostigmine group, respectively — *P* < 0.001).

In patients who underwent open thoracotomy surgery, the median time for TOFR recovery to 0.9 was shorter in the sugammadex group than in the neostigmine group (81 [42–129] s vs. 240 [150–348] — *P* = 0.003). The same difference was found in patients who underwent surgery with video-assisted technique (median time 95 [54–170] s vs. 387 [180–598] s in sugammadex and neostigmine group, respectively — *P* < 0.001).

## Discussion

This multicenter double-blind randomized clinical trial shows that the use of sugammadex shortens the time from reversal agent administration to a TOFR of 0.9 compared to the use of neostigmine. This superiority of sugammadex in terms of efficacy also results in faster extubation times but not in earlier discharge from the operating theater.

The importance of adequate recovery from neuromuscular blockade at the end of anesthesia is related to the need to avoid postoperative residual paralysis by reducing the risk of potentially serious or fatal postoperative respiratory complications [17]. Such recovery can take a long time, so decurization at the end of the procedure is the solution to reduce this waiting period. The greater efficacy of sugammadex compared with neostigmine has been demonstrated in several surgical settings and by the results of two meta-analyses by the Cochrane Collaboration [18, 19]. Limited data exist on patients who undergo thoracic surgery. Our findings contribute to the results of recent studies and are consistent with them. In 2019, Citil et al. published a randomized controlled trial (RCT) on 60 patients undergoing VATS surgery that compared the efficacy of sugammadex and neostigmine [20]. The authors did not report the level of the neuromuscular block at which the reversal agents were administered, but after a maintenance of a deep block during surgery (post-tetanic count between 1 and 2), the mean recovery time in the sugammadex group was shorter than in the neostigmine group (6.1 vs. 22 min, respectively). In 2020, another RCT conducted on 92 patients undergoing VATS surgery reported, as secondary endpoint, a shorter time from the second twitch at the TOF stimulation to a TOFR of 0.9 in patients treated with sugammadex (median time: 10 min) than patients treated with neostigmine (median time: 40 min) [21]. Finally, in 2022, Yu et al. published a RCT on 100 patients who underwent VATS lobectomy reporting again a faster mean recovery time from the third twitch at TOF count to a TOFR of 0.9 of 164.5 s after administration of 2 mg/kg of sugammadex and of 562.9 s after neostigmine [22]. In the present study, this difference is evident for the recovery of TOFR to 0.9 and even sharper for the attainment of 1.0. We also found that the percentage of patients that reaches a TOFR of 0.9 within 5 and 10 min after reversal administration is significantly higher among patients treated with sugammadex than those who received neostigmine.

A more rapid recovery of neuromuscular function at the end of surgery should shorten the time of tracheal extubation. In the present study, the efficacy of sugammadex was superior to that of neostigmine by shortening extubation time by about 6 min. These findings are consistent with previous studies conducted in thoracic patients [20–24] but not resulting in faster discharge of patients from the PACU to the ward. In the thoracic setting, the PACU length of stay has been found to be shorter using sugammadex compared to neostigmine or pyridostigmine by some authors [22, 24] but not by others [21, 25]. Thus, we believe that speeding up the recovery time from residual neuromuscular blockade reduces the time for tracheal extubation and, likely, the operating room occupation time but not the length of stay in the PACU in the absence of a standardized discharge pathways.

Literature not specifically focused on thoracic anesthesia has shown that sugammadex is more effective than neostigmine not only in reducing the recovery time from residual neuromuscular blockade but also in reducing the incidence of adverse events [19]. In the present study, there were no differences in early or late adverse events between the two study groups. More patients reported altered swallowing capability after extubation in the sugammadex group but without reaching a significant difference. Similarly, we did not find difference between the two groups as regard oxygenation after extubation, at PACU discharge, and in the ward. PPCs rate was similar in the two groups and lower than reported by other authors. In fact, we found a PPCs rate around 14% that is significantly lower than recently reported by Yang et al. in a meta-analysis that included seven studies [23]. The authors found a significant difference PPCs rate in favor of patients treated with sugammadex (pooled rate 33%) over patients treated with anticholinesterase inhibitors (pooled rate 47%). To notice, a recent Italian RCT that enrolled 880 patients comparing two different one-lung ventilation strategies reported an overall PPCs incidence of 29.6% [26]. We believe that the lower PPCs rate that we find in our study could be in part related to the fact that all patients were treated with full-dose reversal agents and extubated only after reaching at least a TOFR of 0.9, which did not always happen in the studies considered by the meta-analysis [23].

The sensitivity analysis shows that the use of sugammadex results in faster recovery from all levels of residual neuromuscular blockade. In general, the literature is poor on antagonism of superficial blockade. The protocols of the other studies in the thoracic setting also involved antagonizing neuromuscular blockade from a predefined level (e.g., from the second or third twitch at the TOF stimulation [21, 22]), thus excluding studying patients with very superficial blockade. In our study, it emerges that the superiority of sugammadex over neostigmine also occurs for shallow and minimal levels of residual neuromuscular block. One would expect most patients undergoing thoracic surgery to be managed with a deep level of neuromuscular blockade to avoid diaphragm contractions [8]. This should result in most cases in the finding of a deep or at least moderate residual neuromuscular block at the end of surgery. Nevertheless, in our study, the residual blockade at the end of surgery was shallow or minimal in about 50% of patients. This suggests that anesthesiologists very often do not deliberately aim to maintain deep block during thoracic surgery but lean toward management based on clinical need.

The present study is limited by having been prematurely terminated, and our findings must be interpreted with caution. In particular, this applies to secondary outcomes that could have provided significant information in favor of either study group.

In conclusion, sugammadex enhances the recovery of TOFR to 0.9 more than neostigmine also in patients undergoing thoracic surgery. Sugammadex enables faster recovery of TOFR regardless of the level of residual neuromuscular blockade.

## Supplementary Information

**Additional file 1.**

## Data Availability

The data that support the findings of this study are available from the authors upon reasonable request.

## References

[1] Arbous MS, Meursing AEE, van Kleef JW et al (2005) Impact of anesthesia management characteristics on severe morbidity and mortality. Anesthesiology 102:257–268 15681938 10.1097/00000542-200502000-00005

[2] Della Rocca G, Vetrugno L, Coccia C et al (2016) Preoperative evaluation of patients undergoing lung resection surgery: defining the role of the anesthesiologist on a multidisciplinary team. J Cardiothorac Vasc Anesth 30:530–538 10.1053/j.jvca.2015.11.018 27013123

[3] Stephan F (2000) Pulmonary complications following lung resection. A comprehensive analysis of incidence and possible risk factors. Chest 118:1263–1270 11083673 10.1378/chest.118.5.1263

[4] Bernard A, Deschamps C, Allen MS et al (2001) Pneumonectomy for malignant disease: factors affecting early morbidity and mortality. J Thoracic Cardiovasc Surg 121:1076–1082 10.1067/mtc.2001.114350 11385374

[5] Licker MJ, Widikker I, Robert J et al (2006) Operative mortality and respiratory complications after lung resection for cancer: impact of chronic obstructive pulmonary disease and time trends. Ann Thoracic Surg 81:1830–1837 10.1016/j.athoracsur.2005.11.048 16631680

[6] Agostini P, Cieslik H, Rathinam S et al (2010) Postoperative pulmonary complications following thoracic surgery: are there any modifiable risk factors? Thorax 65:815–818 20805178 10.1136/thx.2009.123083

[7] Murphy GS, Szokol JW, Marymont JH, Greenberg SB, Avram MJ, Vender JS (2008) Residual neuromuscular blockade and critical respiratory events in the postanesthesia care unit. Anesth Analg 107:130–137 18635478 10.1213/ane.0b013e31816d1268

[8] Hemmerling TM, Schmidt J, Hanusa C, Wolf T, Schmitt H (2000) Simultaneous determination of neuromuscular block at the larynx, diaphragm, adductor pollicis, orbicularis oculi and corrugator supercilii muscles. Br J Anaesth 85:856–860 11732519 10.1093/bja/85.6.856

[9] Saitoh Y, Oshima T, Nakata Y (2008) Monitoring of vecuronium-induced neuromuscular blockade during one-lung ventilation. J Anesth 22:378–384 19011776 10.1007/s00540-008-0666-7

[10] Blobner M, Eriksson LI, Scholz J, Motsch J, Della Rocca G, Prins ME (2010) Reversal of rocuronium-induced neuromuscular blockade with sugammadex compared with neostigmine during sevoflurane anaesthesia: results of a randomised, controlled trial. Eur J Anaesthesiol 27:874–881 20683334 10.1097/EJA.0b013e32833d56b7

[11] Geldner G, Niskanen M, Laurila P et al (2012) A randomised controlled trial comparing sugammadex and neostigmine at different depths of neuromuscular blockade in patients undergoing laparoscopic surgery. Anaesthesia 67:991–998 22698066 10.1111/j.1365-2044.2012.07197.x

[12] Jones RK, Caldwell JE, Brull SJ, Soto RG (2008) Reversal of profound rocuronium-induced blockade with sugammadex: a randomized comparison with neostigmine. Anesthesiology 109:816–824 18946293 10.1097/ALN.0b013e31818a3fee

[13] Dindo D, Demartines N, Clavien PA (2004) Classification of surgical complications: a new proposal with evaluation in a cohort of 6336 patients and results of a survey. Ann Surg 240:205–213 15273542 10.1097/01.sla.0000133083.54934.ae PMC1360123

[14] Della Rocca G, Iannuccelli F, Pompei L, Pietropaoli P, Reale C, Di Marco P (2012) Neuromuscular block in Italy: a survey of current management. Minerva Anestesiol 78:767–773 22374378

[15] van Dorn A (2020) COVID-19 and readjusting clinical trials. Lancet 396:523–524 32828180 10.1016/S0140-6736(20)31787-6 PMC7440868

[16] Moher D, Hopewell S, Schulz KF, et al (2010) CONSORT 2010 explanation and elaboration: updated guidelines for reporting parallel group randomised trials. BMJ 340 10.1136/bmj.c869 PMC2844943 20332511

[17] Murphy GS, Brull SJ (2010) Residual neuromuscular block: lessons unlearned. Part I: Definitions, incidence, and adverse physiologic effects of residual neuromuscular block. Anesth Analg 111:120–128 20442260 10.1213/ANE.0b013e3181da832d

[18] Abrishami A, Ho J, Wong J, Yin L, Chung F (2009) Sugammadex , a selective reversal medication for preventing postoperative residual neuromuscular blockade. 10.1002/14651858.CD007362.pub2 19821409

[19] Hristovska AM, Duch P, Allingstrup M, Afshari A (2017) Efficacy and safety of sugammadex versus neostigmine in reversing neuromuscular blockade in adults. Cochrane Database Syst Rev. 10.1002/14651858.CD012763 28806470 10.1002/14651858.CD012763 PMC6483345

[20] Baysal Çitil A, Alıcıkuş Tuncel Z, Yapıcı N, Kudsioğlu T, Aykaç Z (2019) Kavaklı AS (2019) Reversal of rocuronium induced neuromuscular blockade in lung resection surgery: a comparison of sugammadex and neostigmine. GKDA Derg 25:23–30. 10.5222/GKDAD.2019.49369

[21] Moon TS, Reznik S, Pak T, et al (2020) Sugammadex versus neostigmine for reversal of rocuronium-induced neuromuscular blockade: a randomized, double-blinded study of thoracic surgical patients evaluating hypoxic episodes in the early postoperative period. J Clin Anesth 109804 10.1016/j.jclinane.2020.109804 32353805

[22] Yu Y, Wang H, Bao Q, Zhang T, Chen B, Ding J (2022) Sugammadex versus neostigmine for neuromuscular block reversal and postoperative pulmonary complications in patients undergoing resection of lung cancer. J Cardiothorac Vasc Anesth 36:3626–3633 35662514 10.1053/j.jvca.2022.03.033

[23] Yang JL, Chen KB, Shen ML, Hsu WT, Lai YW, Hsu CM (2022) Sugammadex for reversing neuromuscular blockages after lung surgery: a systematic review and meta-analysis. Medicine (United States) 101:E30876 10.1097/MD.0000000000030876 PMC9524927 36181093

[24] Murphy GS, Avram MJ, Greenberg SB et al (2021) Neuromuscular and clinical recovery in thoracic surgical patients reversed with neostigmine or sugammadex. Anesth Analg 133:435–444 33323787 10.1213/ANE.0000000000005294

[25] Song SW, Yoo KY, Ro YS, Pyeon T, Bae HB, Kim J (2021) Sugammadex is associated with shorter hospital length of stay after open lobectomy for lung cancer: a retrospective observational study. J Cardiothorac Surg 16:45 33757525 10.1186/s13019-021-01427-9 PMC7987114

[26] Piccioni F, Langiano N, Bignami E et al (2023) One-lung ventilation and postoperative pulmonary complications after major lung resection surgery. A multicenter randomized controlled trial. J Cardiothorac Vasc Anesth 37:2561–2571 37730455 10.1053/j.jvca.2023.04.029 PMC10133024

