# Supplementary material for: Decurarization After Thoracic Anesthesia using sugammadex compared to neostigmine (DATA trial): a multicenter randomized double-blinded controlled trial
Source: J Anesth Analg Crit Care. 2024 Feb 8;4:9. doi: 10.1186/s44158-024-00146-6 (PMC10854138; doi:10.1186/s44158-024-00146-6)
Supplement: Supplementary file 1 — Additional file 1. [file 44158_2024_146_MOESM1_ESM.pdf]

Additional file

Decurarization After Thoracic Anesthesia using sugammadex compared to neostigmine (DATA trial). A multicenter randomized double-blinded controlled trial

Anesthesia protocol 2

TOF-Watch Sx calibration protocol 3

Definition of complications 4

Details on Trial Interruption 5

Anesthesia protocol

On arrival in the operating room, a thoracic epidural catheter was placed according to local Acute Pain Service protocol. Patient monitoring included electrocardiogram, pulse oximetry, end-tidal carbon dioxide, airway pressures, invasive blood pressure, neuromuscular function monitoring, esophageal temperature.

General anesthesia induction was performed using propofol, remifentanil and rocuronium bromide. A double lumen tube (DLT) or a single lumen tube with bronchial blocker (BB) were positioned and checked with bronchoscopy. The patients were warmed using a forced air warming blanket and a fluid warmer, if needed. Anesthesia was maintained with remifentanil and inhaled anesthetics or intravenous propofol accordingly to the anesthesiologist's choice.

During two-lung ventilation the tidal volume was set to 8 ml/kg, FiO_2=0.5, inspiratory time to 33%, the respiratory rate (RR) was adjusted to maintain the end-tidal CO_2 value < 40 mmHg, and positive end-expiratory pressure (PEEP) was set to 5 cmH_2O. During OLV the tidal volume was decreased to 4-6 ml/kg and RR adjusted to maintain the end-tidal CO_2 < 50 mmHg. At the end of the procedure, after secretions suctioning, an alveolar recruitment maneuver was performed as follows: the ventilator was set in a pressure-controlled modality with an inspiratory pressure of 30 cmH_20, the inspiratory time to 50%, RR set to 10 per minute and PEEP = 5 cmH_2O for 10 breaths.

Intravenous paracetamol 1 gr and ketorolac 30 mg were administered 30 minutes before the end of the procedure.

TOF-Watch Sx calibration protocol

Eight steps:

1. Placement of two pediatric electrodes 3-4 cm apart on the course of the ulnar nerve near the wrist of the limb contralateral to the surgical site
2. Application of accelerometric sensor to the thumb employing the appropriate Hand Adapter device (Organon Teknika BV, Boxtel, Holland)
3. Application of 10 single twitch stimuli at 1Hz using an intensity of 50 mA verifying the correct motor response to the electrical stimulus
4. Application of tetanic stimulation at 50Hz for 5 seconds
5. Calibration of the device using the CAL2 algorithm
6. Start of continuous stimulation at a frequency of 1Hz
7. If twitch values (T1) were maintained within the 95-105% range after 3 minutes, rocuronium could be administered
8. If T1 values deviated from the 95-105% range, it was necessary to recalibrate the device starting again from "step 5"

Definition of complications

Respiratory infection was defined as the appearance of a new infiltrate on chest radiograph combined with at least two of the following criteria: body temperature greater than 38° C or less than 35.5° C, white blood cell count greater than 12000/mm^3 or less than 4000/mm^3, presence of purulent sputum.[1]

Respiratory failure was defined by the occurrence in the postoperative period of an SpO_2 less than 90%, or a PaO_2/FiO_2 ratio less than 300,[2] a $PaCO_2$ greater than 45 mmHg,[2] the onset of dyspnea with respiratory fatigue and/or use of the accessory muscles of respiration.

Pleural effusion was identified as the appearance in the postoperative period of a glaze on chest radiograph not present on previous radiograms or by the identification by ultrasonography of a collection of non-corpuscular fluid in the pleural cavity.[3] Pleural effusion can also be defined by a hypo-/anechogenic zone between the visceral and parietal pleura.[4]

The presence of pneumothorax was defined as the presence of air in the pleural space diagnosed by chest radiography[5] or by ultrasound imaging showing loss of "gliding sign" or "lung sliding," referring to movement related to breathing.[4]

Pneumothorax was defined as the presence of air in the pleural space on chest X-ray or CT scan or the loss of the "gliding sign" or "lung glide," which refers to breath-dependent upward and downward movement on ultrasound examination.[5]

Atelectasis was defined as opacification of a lobe or lobar segment in question. With a compensatory overinflation of the remaining aerated segments in the affected lobe.[6]

Aspiration pneumonia was defined as acute lung injury following inhalation of regurgitated gastric contents.[7]

Bronchospasm was defined as a newly detected expiratory wheeze and treated with bronchodilators.[1]

References:

1. Ferrer M, Liapikou A, Valencia M. Validation of the American Thoracic Society-Infectious Diseases Society of America guidelines for hospital-acquired pneumonia in the intensive care unit. Clin Infect Dis 2010; 50:945-52.
2. Boldrini R, Fasano L, Nava S. Noninvasive mechanical ventilation. Curr Opin Crit Care 2012;18:48-53.
3. Maskell NA, Butland RJ. BTS guidelines for the investigation of a unilateral pleural effusion in adults. Thorax 2003;58:8–17.
4. Reissig A, Copetti R, Kroegel C. Current role of emergency ultrasound of the chest. Crit Care Med 2011;39:839-45.
5. Henry M, Arnold T, Harvey J. BTS guidelines for the management of spontaneous pneumothorax. Thorax 2003;58:39–52.
6. Duggan M, Kavanagh BP. Pulmonary atelectasis: A pathogenic perioperative entity. Anesthesiology 2005;102:838–54.
7. Marik PE. Aspiration pneumonitis and aspiration pneumonia. N Engl J Med 2001;344:665–71.
8. Canet J, Gallart L, Gomar C et al. Prediction of postoperative pulmonary complications in a population-based surgical cohort. Anesthesiology 2010;113:1338-50.

Details on Trial Interruption

The recruitment of patients was prematurely terminated in 2020 by the scientific committee because it was deemed very difficult to reach the established sample size due to the overall slow recruitment rate and the onset of the COVID-19 pandemic.

A post-hoc Conditional Power was calculated assuming that the mean difference in the primary outcome between the two study groups (time from reversal administration to at least 3 train-of-four ratio values = or > 0.9) was due to the observed trend. The analysis was performed with the LongCART package in a R shiny app (freely available at https://ppos.shinyapps.io/public/)[1].
The analysis result is reported below:

	Expected mean difference between study groups	Observed mean in the neostigmine group	Observed mean in the sugammadex group	Observed mean difference between study groups	Conditional Power for clinical success*	Conditional Power for trial success°
DATA trial	300 sec (= 5 min)	543 (SE 115)	99 (SE 16)	431 (SE 116)	99.5%	74.2%

SE, standard error; * Achieving threshold of 300 sec; ° Achieving statistical significance at 1-sided 0.05 level

A post-hoc power analysis was also performed on the same data using a one tailed t-test with G*Power 3.1 software[2] (freely available at https://www.psychologie.hhu.de/arbeitsgruppen/allgemeine-psychologie-und-arbeitspsychologie/gpower). The results are reported below:

Alpha-error probability (1 tail): 0.05
Effect size: 0.902
Power (1 - Beta-error probability) = 0.981 (98.1%)

Distributions plot:

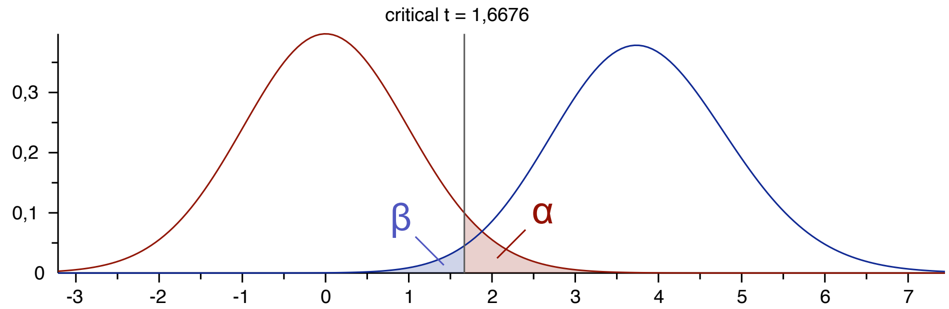

[1] Kundu, M.G., Samanta, S. & Mondal, S. (2023) Review of calculation of conditional power, predictive power and probability of success in clinical trials with continuous, binary and time-to-event endpoints. *Health Serv Outcomes Res Method*. https://doi.org/10.1007/s10742-023-00302-5
[2] Faul, F., Erdfelder, E., Buchner, A., & Lang, A.-G. (2009). Statistical power analyses using G*Power 3.1: Tests for correlation and regression analyses. *Behavior Research Methods*, *41*, 1149-1160

Plot of power vs. sample size:

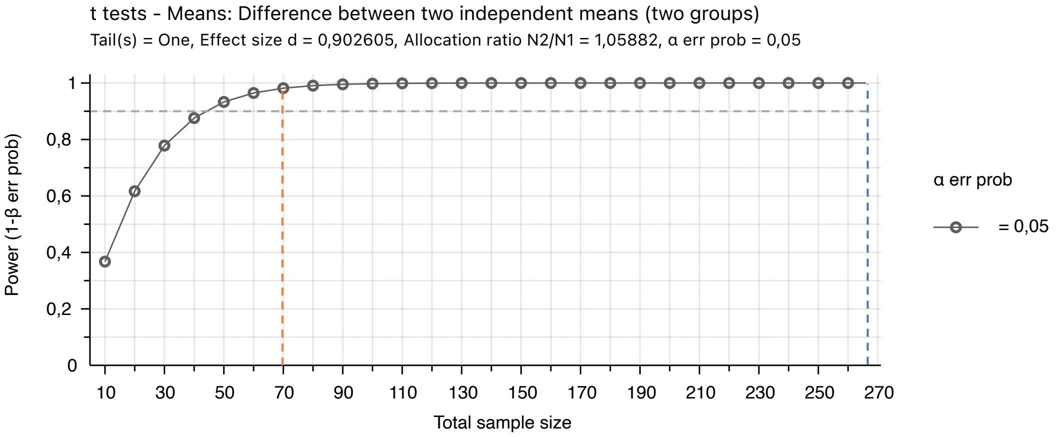

[Grey line: power of 90%; orange line: number of recruited patients; blue line: planned sample size]

Given the observed mean difference between the study groups (431 sec), which was higher than expected, and both high conditional power values and high post-hoc estimated power of the trial, the Steering Committee considered the decision to discontinue the study to be correct. If enrollment continued to 266 cases, it was highly unlikely to observe a statistically significant difference in the primary endpoint compared to the results obtained from the analysis of the recruited patients.
